# Delivering youth nutrition interventions through school-based gardening of indigenous vegetables and fruits and WhatsApp nutrition education in Southwest Nigeria: non-randomized study protocol

**DOI:** 10.3389/fnut.2025.1539861

**Published:** 2025-06-02

**Authors:** Abiodun T. Atoloye, Idowu A. Atoloye, Sherif O. Olasoji, Victoria A. Tanimonure, Michael O. Awoleye, Cornelius T. Atere, Tolulope L. Owoyemi, Atanda S. Oladejo

**Affiliations:** ^1^Department of Nutrition, Dietetics, and Food Sciences, Utah State University, Logan, UT, United States; ^2^Department of Agriculture, Alcorn State University, Lorma, MS, United States; ^3^Department of Plants, Soil, and Climate, Utah State University, Logan, UT, United States; ^4^Department of Agricultural Economics, Obafemi Awolowo University, Ile-Ife, Nigeria; ^5^African Institute of Science Policy and Innovation, Obafemi Awolowo University, Ile-Ife, Nigeria; ^6^Department of Soil Science and Land Resources Management, Obafemi Awolowo University, Ile-Ife, Nigeria; ^7^Horticulture Nigeria Project, Obafemi Awolowo University, Ile-Ife, Nigeria; ^8^Department of Crop Production and Protection, Obafemi Awolowo University, Ile-Ife, Nigeria

**Keywords:** youth nutrition, school-based gardening, WhatsApp-based nutrition education, indigenous vegetables and fruits, dietary behaviors, nutrition-related outcomes

## Abstract

**Introduction:**

The poor dietary habits and limited nutritional knowledge, particularly regarding indigenous vegetables and fruits (IVFs) among youth in southwest Nigeria, highlight the need for an integrated intervention approach. Integrating school-based gardening focused on IVFs with a nutrition education program delivered via WhatsApp combines experiential learning with digital tools.

**Methods and analysis:**

This is a non-randomized, mixed-methods study involving youths between 15 and 35 years who will participate in gardening activities and/or interactive nutrition education via WhatsApp. Participants will be provided technical support on growing IVFs, while nutrition education messaging via WhatsApp will include texts, images, and videos on nutrition and healthy eating behavior. Data at baseline will be collected on the study’s primary outcomes (awareness and interest in IVFs, household food security, nutritional knowledge and practices, fruits and vegetables intake, food safety self-efficacy, dietary diversity, anthropometric, and biomarker indicators). In contrast, data collection during the intervention and at post-intervention will include the study’s secondary outcomes (WhatsApp engagement, knowledge retention, and intervention acceptability). Mixed model regression and the Mann–Whitney U Test will be used to analyze the data collected. All analyses will be performed using IBM SPSS (version 23), and the statistical significance will be set at a *p*-value <0.05.

**Discussion:**

The present study will focus on the acceptability and feasibility of gardening and incorporate nutrition education delivered through WhatsApp to address the improvements in food security, dietary diversity, and other nutrition-related outcomes of youth in low-income countries. The expected outcomes include enhanced nutrition knowledge, healthier dietary habits, and greater acceptance of indigenous gardening. The result will support the development of effective, culturally acceptable strategies to promote healthy eating behavior among youths and influence future school-based nutrition programs in similar settings.

## 1 Introduction

Youths, also known as “young adults,” are generally referred to as individuals in the developmental stage between childhood and adulthood (1). Globally, youths are a focus of public health and educational initiatives due to their potential for long-term impact on future health and well-being. Youths in Nigeria are all young people between the ages of 15 and 35 years (2). Nigeria has one of the largest youth populations in the world, with a median age of 18.1 years, and about 70% of the population is under 30 years of age (3). Previous cross-sectional studies conducted among Nigerian youths reported low nutritional knowledge and inadequate dietary habits (4), and high intake of starchy foods, cereals, legumes, and sugar with a variance of 11.5% (5). Furthermore, a recent systematic review study conducted among Nigerian youths reported a low intake of fruits and vegetables (6). In addition, cross-sectional studies have shown poor fruit and vegetable consumption among Nigerian youth. For instance, 32.4% of participants consumed infrequent fruits and vegetables intake, while only 9.7% of Nigerian youth met the recommended daily intake of fruits and vegetables (4). Furthermore, food insecurity rates among this age group have been reported to range between 15 and 48% (5, 7–10). The combination of these nutrition-related problems among this age group makes them an important target group for nutritional interventions.

The efficacy of nutrition interventions such as school-based feeding programs, community gardening, and nutrition education has been established in high-income countries (11). However, their efficacy among youths in low-middle-income countries such as Nigeria remains underexplored and less widely disseminated (12). Of these interventions, community gardening of IVFs presents a valuable opportunity to address food insecurity, promote diversity, preserve cultural food heritage, and improve dietary diversity and nutrition by providing locally available, nutrient-dense foods (13–16). Furthermore, the intervention may promote sustainability and local agriculture, reduce dependence on imported food, and deepen youth engagement with their local environment (17–19).

In recognizing the significance of indigenous food systems, the Task Force on Traditional and Indigenous Food Systems and Nutritional Sciences (IUNS) has emphasized the need for increased public awareness and further research into the benefits of IVFs (20). While many studies have highlighted low nutritional knowledge among youths and recommended an increase in IVF consumption and participation in vegetable gardening (21–24), most of these studies are cross-sectional, which prevents legitimate causal inferences and limits the prospects of studying the effectiveness of an intervention on nutrition outcomes in youth. This highlights the need for intervention research that promotes utilization and evaluates the effectiveness of IVFs on nutritional outcomes in youth. Moreover, previous studies have recommended implementing multiple interventions to improve nutrition and health outcomes (13, 25).

The integration of nutrition education in school-based IVFs gardening among youths holds promise for enhancing dietary habits and overall nutrition among the young population. Given the youth’s widespread use of mobile technology and its potential for delivering educational content, digital platforms could enhance these interventions. WhatsApp remains one of the easily accessible mobile phone messaging applications in Nigeria due to its broad usage among educators, community health workers, and students (26, 27). Various studies have proven that engaging youths in health education through WhatsApp is effective (28, 29). While a few studies have explored text messaging platforms for delivering nutrition education among Nigerian youths (30), none have yet integrated a WhatsApp-based nutrition education component into school-based IVF gardening programs. WhatsApp is widely adopted and offers advantages over traditional/face-to-face nutrition education. This may be because the former permits the inclusion of multimedia such as images, videos, audio, and document files, making it a more versatile and engaging tool for communication and education (31–33). Exploring the integration of digital platforms with hands-on experiential learning could present an opportunity to improve youth nutrition, particularly in areas with limited access to traditional interventions (34). Hence, this study will utilize WhatsApp as a digital learning tool to achieve the purpose of this study and will thereafter measure its impact on the target audience.

Specifically, the study will assess the impact of a school-based gardening program and WhatsApp-based nutrition education interventions on primary outcomes such as youths’ nutrition knowledge, fruit and vegetable-specific self-efficacy, food security, dietary diversity, anthropometric data, and skin carotenoid level. In this study, school-based gardening refers to structured gardening activities conducted within a school setting involving in-school and out-of-school youths residing in the same community as the school. These activities will engage participants in cultivating and harvesting vegetables to enhance agricultural skills, nutrition awareness, and healthy eating habits. This intervention will be guided by the Cognitive Theory of Multimedia Learning (CTML), Social Cognitive Theory (SCT), and Empowerment Theory (ET), all of which are described in details in the method section below. Furthermore, the study will also examine secondary outcomes such as the participants’ engagement, knowledge retention, and acceptability of the intervention.

## 2 Methods and analysis

### 2.1 Study aim

This study aims to assess the feasibility and effectiveness of the delivery of nutrition interventions through combined school-based gardening of IVFs and WhatsApp nutrition education among the youth in Southwest Nigeria. Therefore, we hypothesize as follows:

**Null Hypothesis:** *There is no difference in nutrition knowledge, fruit and vegetable self-efficacy, dietary habits, and biochemical data between youth who receive the combination of school-based gardening of IVFs, and WhatsApp nutrition education compared to those who receive only school-based gardening of indigenous fruits and vegetables.*

**Alternative Hypothesis:** *There is a significant difference in nutrition knowledge, fruit and vegetable self-efficacy, dietary habits, and biochemical data between youth who receive the combination of school-based gardening of IVFs and WhatsApp nutrition education compared to those who receive only school-based gardening of indigenous fruits and vegetables.*

### 2.2 Overview and study design

This research design for the study is a non-randomized intervention study (quasi-experimental study design). A non-randomized study design was selected due to some feasibility constraints and logistical challenges. This includes challenges with schools meeting the inclusion criteria, such as the requirement for arable land, access to water facilities, and the presence of agricultural and nutrition teachers. Additionally, to align with policy priorities, ensure feasibility and sustained participation, sites will be selected in collaboration with state and local education authorities as well as community leaders, making random assignment impractical.

**Figure 1:**
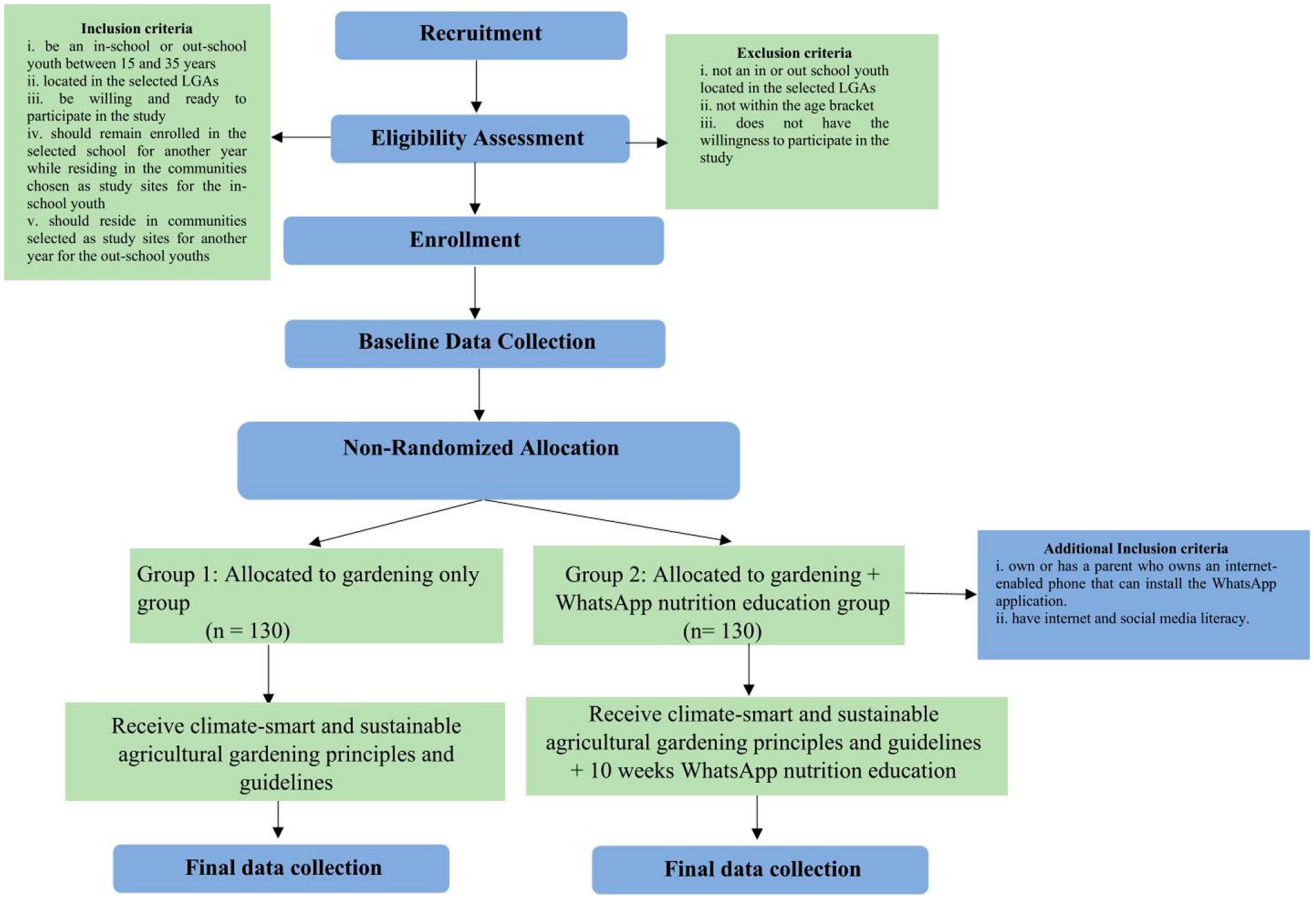
Study flowchart.

The study will be carried out in three Southwest Nigeria states which were selected from six states in the region: Ekiti, Osun, and Ogun. There will be two arms of intervention; one group will receive the school-based gardening of IVFs, and the second group will receive both school-based gardening of IVF and WhatsApp nutrition education. This means all participants will participate in the school-based gardening program of IVFs intervention. In addition, 50% (*n* = 130) of the participants will be selected for the nutrition education based on pre-selected criteria such as access to a web-supported phone and knowledge using social media like WhatsApp. Figure 1 summarizes the flow of activities in the study.

#### 2.2.1 Sampling technique and sampling size

Our target population consists of unemployed youths, with estimates ranging between 6.8 and 35% (35–38). Since this is a first of its kind study with no prior effect size for power calculation and known prevalence of IVFs consumption among the target population, a conservative sample size calculation approach was used (39). The sample size calculation applied the population proportion of 18.8% (the percentage of the unemployed Nigerian youth at 2020) in its estimate as shown below (35, 37). The sample size of 235 was calculated at a 95% confidence level and a margin error of 5% using an online calculator (40). Using a 10% expected dropout. The sample size is approximately 260 (130 per group). The formula used is shown below:


$$n=\frac{z^{2}\times \hat{p}(1-\hat{p})}{\varepsilon^{2}}$$

Where:

n is the sample size

z score (1.96 for 95% confidence level)

ε is the margin of error (0.05)

p̂  is the population proportion (0.188)

Study participants will be selected using a multi-stage sampling technique, that will incorporate non-randomized, purposive sampling technique at the state, within the state, schools/community, and individual levels. The details below describe how the estimated sample size will be achieved with the sampling technique.

#### 2.2.2 Participants recruitment

Youths aged 15–35 will be identified from schools (in-school youths) and communities (out-of-school youths) in the selected states mentioned above. The selection of target states was based on purposive selection, considering their commonality in the IVFs of interest. The second stage will involve the selection of participants from each of the six selected communities (one from a rural and another from an urban local government area (LGA) in each of the selected states). LGA is an administrative division within a Nigeria that functions as the third tier of government, below the national and state levels. The target rural and urban LGA will be randomly selected from each state’s list of LGAs.

The selection of target schools will be facilitated in collaboration with the leadership of the State Ministry of Education, based on pre-selected criteria that can support onsite gardening activities. These criteria include having: a piece of land suitable for farming, access to water, and either a fenced perimeter or good security measures in place. After the school is identified, the researcher will collaborate with school administrators and teachers in the selected communities to recruit eligible in-school and out-of-school youths. Out-of-school youths will be selected from the communities where the selected schools are situated to bridge the gap between the schools and the wider community, ensuring that the gardening projects are inclusive, sustainable, and community-driven. The summary of eligibility criteria for youth selection for the gardening intervention is highlighted as follows:

- Be an in-school or out-school youth between 15 and 35 years.
- Located in the selected LGAs.
- Should remain enrolled in the selected school for another year while residing in the communities chosen as study sites for the in-school youth.
- Should reside in communities selected as study sites for another year for the out-of-school youths.

The exclusion criteria include:

- Not an in or out-of-school youth located in the selected LGAs.
- Not within the age bracket (15–35 years).
- Does not have the willingness to participate in the study.

Youths that would be enrolled in the nutritional education arm of the intervention should:

- Own or has a parent who owns an internet-enabled phone that can install the WhatsApp application.
- Have internet and social media literacy

The exclusion criteria for the nutrition education arm of the intervention are:

- Does not own or have good access to the parent’s web-based phone that supports the installation of the WhatsApp application
- Does not have internet and social media literacy
- Not willing to participate in the intervention program

### 2.3 Theoretical basis

The Cognitive Theory of Multimedia Learning (CTML), Social Cognitive Theory (SCT), and Empowerment Theory (ET) will inform the intervention. CTML informs the development of effective multimedia messages engaging working memory through two learning channels-verbally and pictorially, enabling readers to construct mental links among visual and textual representations (41). Participants would be required to fuse the new information with the previous knowledge to create a mental representation for the information to be retained (41), activating both verbal and pictorial learning models for the participants will create a piece of integrated information in the working memory through active processing.

**Figure 2:**
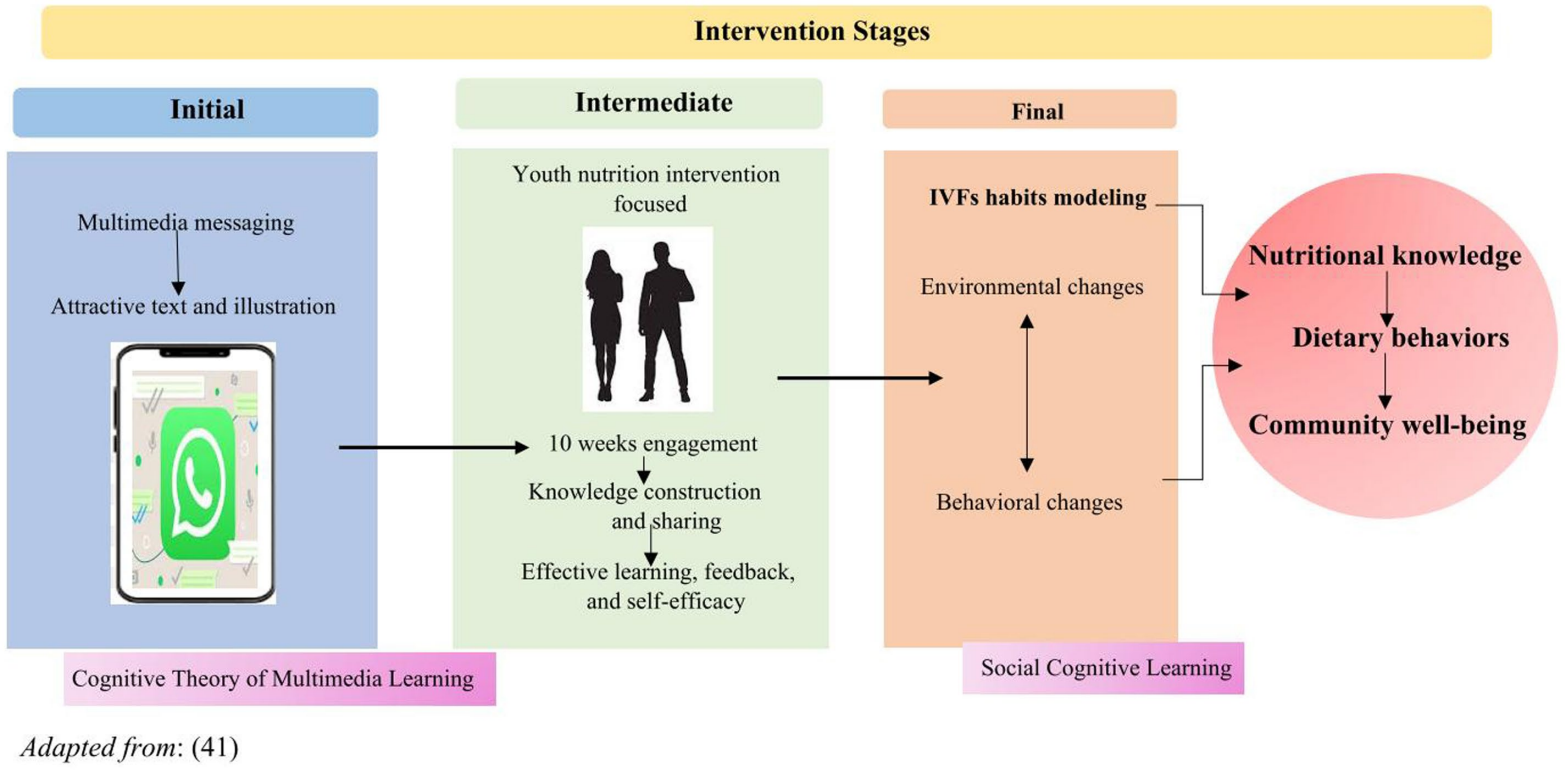
Intervention framework for the WhatsApp nutrition education component of the study.

Given the focus on social cognitive outcomes such as nutrition knowledge and vegetable and fruit-specific self-efficacy in this study, the SCT will be utilized. This theory posits that human behavior results from the interactions between personal factors, environmental influences, and behavioral patterns. In addition to addressing personal and behavioral outcomes on SCT, this study will also consider relevant environmental factors identified in previous studies as barriers to the use of IVF innovations. These factors include inadequate funding, lack of access to inputs, institutional support, and community influences (42, 43). Also, the study will use ET, which is a social work theory that helps people gain control and autonomy in their lives (44, 45). Our study will include individual-empowered outcomes such as self-efficacy, skill building, access to resources, or resource mobilization skills. Others will include community involvement and institutional participation. Figure 2 summarizes the relationship between CMLT, SCT, and ET principles, highlighting three key aspects: psychological, community, and social empowerment. This framework specifically focuses on the participants in Group 2, who were involved in climate-smart school-based gardening and WhatsApp nutrition education.

#### 2.3.1 Intervention plan

**Figure 3:**
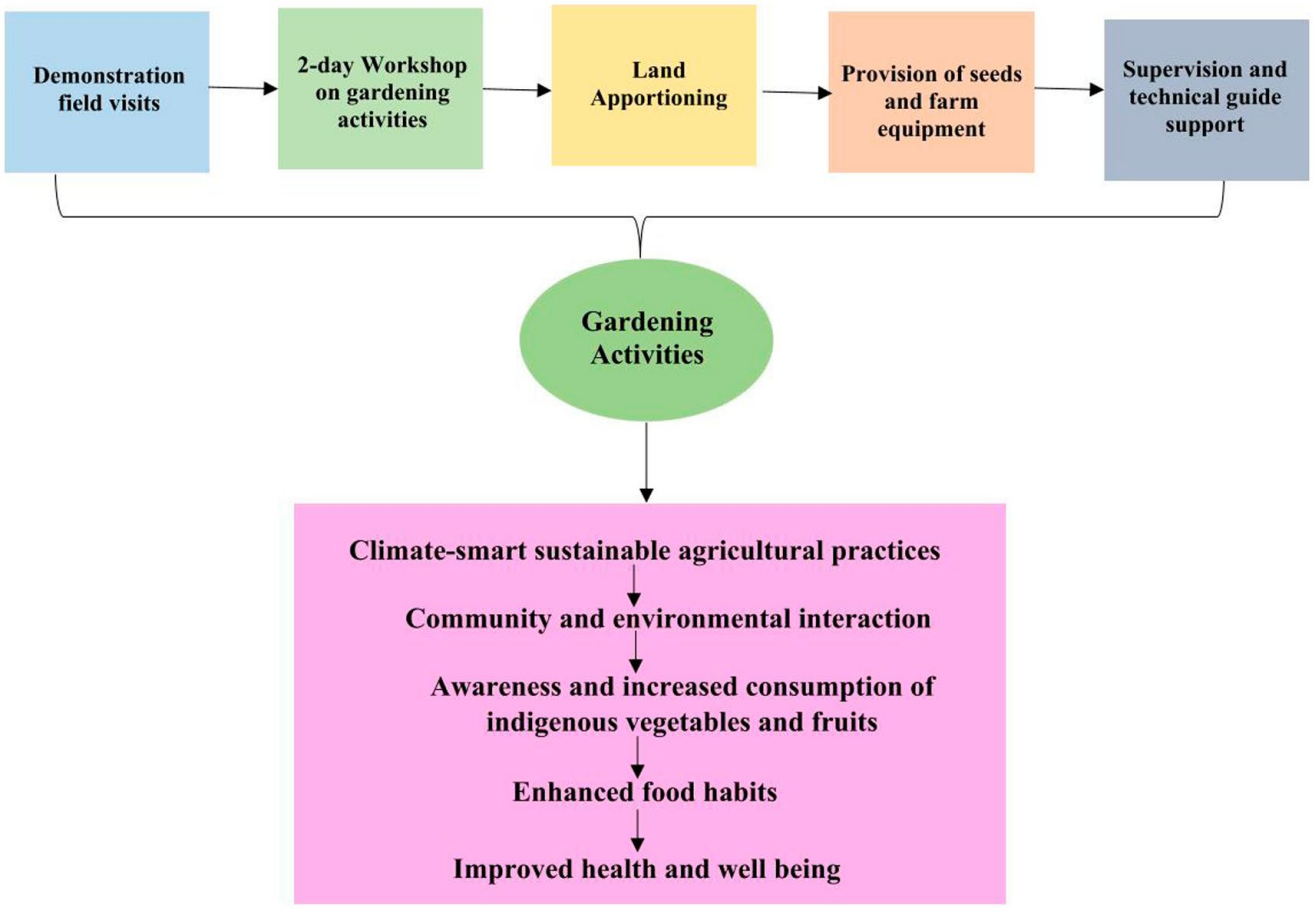
Intervention framework for the school gardening component of the study.

The school-based gardening component of the intervention, as illustrated in Figure 3, will kick off with a 2-day workshop among the targeted participants. This workshop will cover topics such as an introduction to IVF farming and seed production process, showcasing the best soil management practices, and nutrition and food safety education. The participants will also be introduced to digital tools in agriculture, particularly the use of virtual assistants to facilitate sustainable and efficient farming practices, streamlining workflows and revolutionizing how young farmers access expert-level advice virtually, at their convenience. They will also educate participants on agribusiness models for farming and farming-related commercial activities. After the workshop, the youths will be apportioned equal plots for farming on school farmland. Through the collaboration of the school authorities and communities, the out-of-school youths will also have plots to use on the school farmland. The participants will be provided with viable seeds and other farm equipment, such as drips, knapsacks, soil improvement materials etc. to facilitate their farming activities. This will give them the opportunity to utilize the knowledge of IVF farming learned during the workshop. The agricultural science teachers in the selected schools will supervise the new farmers’ work to support community ownership. Furthermore, participants will be provided with technical support to guide them on good soil health management using the climate-smart approach throughout the farming year. Technical support will be provided through both physical and virtual approaches. At the initial stage of transplanting, the technical support will be provided weekly and then fortnightly till the vegetables are well established. Specifically, support will be provided physically to farmers in rural or semi-urban areas who have limited access to internet facilities and electricity, ensuring compliance. In contrast, urban participants will receive support through both physical and virtual means. Leveraging virtual support technology will help save costs and reduce risks associated with city traffic.

Participants selected for the WhatsApp nutrition education will be exposed to the nutrition education curriculum; *Roots and Greens: A Youth Guide to Indigenous Foods and Nutrition (Roots and Greens Curriculum)* for 10 weeks. The curriculum will educate them about the values and uses of indigenous vegetables and fruits. Table 1 describes the content of the *Roots and Greens Curriculum*. The nutrition education intervention involves the use of documents guide, furnished relevant infographics, images, and short video clips. Videos are necessary to make learning interesting and concepts easy to comprehend, which has the propensity to save time reading large texts. The efficacy of video to enhance learning has been widely reported in extant literature (44, 45). This aspect of the intervention will apply interactive tools to create a supportive environment that educates and empowers youth towards sustainable diets, inclusive of IVFs, through quizzes, polls, and group discussions. Table 2 shows the timeline of the study activities according to the intervention stages.

**Table 1:** *Roots and Greens* curriculum for a 10-week WhatsApp nutrition education.

Week	Topics	Objectives	Activities	Tasks
1.	Introduction to healthy eating	Introduce the concept of healthy eating and familiarize students with indigenous vegetables and fruits (IVFs)	Introduction to healthy eating including an activity on creating a balanced meal plate using provided food options that feature IVFs	Participants will be asked to find a recipe that includes IVFs and share in the group chat
2.	Introduction to IVFs	Familiarize students with indigenous vegetables and fruits.	Identification of commonly known indigenous vegetables and fruits by difference in taste, availability, and perceived nutritional values.	Participants will be asked to cook with an indigenous vegetable at home, document the process with photos and a brief description, and share their experience in the group chat
3.	Nutritional benefits of IVFs	Deepen understanding of the nutritional benefits of IVFs	Discussion of food photos from week 2 task and introduction to the health benefits of IVFs	Participants will be asked to share one indigenous vegetable or fruit they ate this week and mention one health benefit they know about it and share same in group chat
4.	Cultivation and harvesting of IVFs	Teach participants how to grow and harvest IVFs	Introduction to gardening and caring for the plants	Participants will be asked to keep a gardening journal and document the growth and care of their plants
5.	Cultural significance and traditional uses of IVFs	Explore the cultural significance and traditional uses of IVFs	Cultural storytelling and guest speaker session	Participants will be asked to share family traditions or recipes that include IVFs in the group chat
6.	Food safety practices	Understand the importance of food safety and best practices	Introduction to food safety, identifying food safety hazards, and safe handling and preparation of IVFs	Participants will be asked to create a food safety checklist and share same in the group chat
7.	Health benefits and disease prevention	Discuss the health benefits of consuming IVFs nd their role in disease prevention	Health benefits overview and role in disease prevention	Participants will be asked to share personal or family stories where diet changes particularly incorporating IVFs fruits have positively impacted health
8.	Putting it all together: cooking and meal planning	Integrate knowledge to cooking and meal planning with indigenous foods	Cooking techniques, meal planning, and cooking activity	Participants will be asked to implement the meal plan and report back on any challenges
9.	Indigenous foods and sustainability	Understand the role of indigenous foods in sustainable eating practices	Sustainability overview, interactive activity, and group project activity	Participants will be asked to implement a sustainable practice at home and report back any challenges
10.	Review and celebration	Review key concepts and celebrate achievements	Review quiz	Participants will be asked to record (audio) of a reflection of their experience about *Roots and Greens*, include what they have learned and how they have applied it and share it on the group page

**Table 2:** Study activities and outcome measures according to the stages.

Stage of intervention	Recruitment	Baseline	Intervention period	Post-intervention evaluation
Verbal consent for screening	√			
Eligibility screening	√			
Study invitation	√			
Participants consent		√		
Goal setting for the groups			√	
2-day workshop on gardening activities			√	
Land apportioning for gardening activities			√	
Provision of seeds and farm equipment			√	
Supervision of gardening activities			√	
Technical guide support for the gardening activities			√	
Multimedia messaging for WhatsApp education intervention group			√	
Tracking WhatsApp education participants’ engagement and knowledge retention			√	
Participants’ socio-demographic characteristics		√		√
Participants’ awareness and interest in indigenous vegetables and fruits		√		√
Participants’ household food security		√		√
Participants’ nutrition knowledge and practices		√		√
Participants’ fruits and vegetables specific self-efficacy		√		√
Participants’ food safety self-efficacy and practices		√		√
Participants’ dietary diversity		√		√
Participants’ anthropometric measures and biomarker data collection		√		√

### 2.4 Participants information and outcome of measures

Using surveys administered by trained enumerators, information about participants’ socio-demographics and the outcomes of measures includes knowledge of nutrition, dietary habits, food security, anthropometrics and biomarker indicators will be obtained at baseline and post-intervention. Acceptability of the intervention will be included in the post-intervention survey. Information about their engagement, and knowledge retention intervention will be tracked during the WhatsApp nutrition education delivery. Table 2 describes the study activities in details.

The validity and reliability of the tools used to measure study outcomes is described below. All adapted and newly developed tools described below will be assessed for internal consistency by calculating Cronbach’s Alpha to determine the correlation between items measuring each construct.

#### 2.4.1 Primary outcomes

##### 2.4.1.1 Awareness and interest in indigenous vegetables and fruits

Given the novelty of this outcome, we developed a six-item questions that cover questions about participant’s awareness of IVFs, interest in IVF gardening, nutritional benefits, barriers to consumption, and their role in enhancing health and well-being were developed with input from agricultural experts for content validity. This measure will assess improvement in awareness and knowledge of IVFs resulting from their participation.

##### 2.4.1.2 Household food security

The study population’s household food security will be measured by using Household Food Insecurity Access Scale (HFIAS). This tool was developed by the Food and Nutrition Technical Assistance Program of the US Agency for International Development has demonstrated validity and reliability in low- and middle-income countries (46). The HFIAS consists of nine occurrence questions targeted at the food access experience of the participants. Each question has 3 responses, namely, rarely (once in the past 4 weeks), sometimes (three to ten times in the past 4 weeks), and often (more than ten times in the past 4 weeks), with scores of 1, 2, and 3, respectively. The scores will be summed to a maximum of 27. The HFIAS will be considered as a continuous variable with higher scores indicating greater susceptibility to food insecurity (46).

##### 2.4.1.3 Nutritional knowledge and practices

A four-item question, one about their knowledge of five-a-day recommendation and three about participants’ nutritional knowledge and practices on IVF will assess the change in nutritional knowledge and practices between pre- and post-intervention. These nutrition practice questions were adapted from the validated food behavior checklist (47).

##### 2.4.1.4 Indigenous vegetables and fruits specific self-efficacy

A five-item question will assess the participants’ confidence in the selection, preparation, and consumption of IVFs. These questions were adapted from a previously validated 24-item self-efficacy consumption of fruit and vegetable (48), and food safety psychosocial questionnaire (49) to capture changes in self-efficacy related to the choice, preparation, and consumption of IVFs.

##### 2.4.1.5 Food safety self-efficacy and practices

A six-item question on food safety self-efficacy was adapted from the food safety self-efficacy construct of a previously food safety psychosocial questionnaire that had demonstrated validity and internal consistency (49). The set of questions will measure the participants’ confidence in practicing safe methods of handling, storing, and preparing food. A descriptive statistics test will be used to determine the participants’ self-efficacy practice levels of food safety at the pre-and post-intervention.

##### 2.4.1.6 Household dietary diversity

The household dietary diversity score (DDS) is a validated proxy measure that reflects a household’s economic ability to access a variety of foods (50). It has demonstrated good content and construct validity, and has been applied reliably across multiple cultural and socioeconomic contexts. It assessed household food access using a structured scoring system based on the consumption of various food groups by participants (51). The assessment shall be made on the food categories of cereals and grains: bread, rice, noodles, biscuits, millet, maize, etc., roots and tubers; eggs; fish and shellfish; legumes and nuts; dairy products, such as milk, cheese, yogurt; fats and oils; sugars and honey; and miscellaneous foods, including condiments, coffee, and tea. Scores shall be given as 1 point for every category taken and 0 for those not taken. In measuring the cumulative DDS, the food items across all food groups will be summed; hence, the maximum score possible will be 12. The higher the dietary diversity score, the higher the dietary diversity. A descriptive statistic of the dietary diversity of the participants, using the score threshold of 0–4 for low dietary diversity, 5–8 for medium dietary diversity, and 9–12 for high dietary diversity scores will be used (10).

##### 2.4.1.7 Anthropometric measurement and biomarker Indicator

Study participants will be assessed on their height (m) and weight (kg) to determine their Body Mass Index (BMI). WHO Anthro software will be used to assess the anthropometric measurement of participants between (15–18 years old) using the BMI for Age metric; the anthropometric measurement of participants between the age of 19 and 35 will be based on WHO standard of underweight if BMI is <18.5 kg/m^2^, normal or healthy weight if BMI is 18.5 kg/m^2^–24.9 kg/m^2^, overweight if BMI is 25–29.9 kg/m^2^, and obese if BMI ≥ 30.0 kg/m^2^ (52). The participants’ skin carotenoid levels will be assessed using the Veggie Meter to assess their fruits and vegetables intake (53).

#### 2.4.2 Secondary outcomes

##### 2.4.2.1 WhatsApp engagement

The study participants’ engagement will be evaluated using WhatsApp metric indicators, such as the messages read counts, the number of messages replied to, the engagement ratio (this will be engagements requiring replies/responses or actions), the rate of tasks completed, responses to surveys and polls, and the participants’ comments and feedback during the discussion. These metrics are part of the standard metrics used to measure the efficacy of WhatsApp’s use for learning (54, 55). The WhatsApp engagement will be tracked and measured using a combination of manual monitoring and WhatsApp’s inbuilt metrics. Precisely, engagement will be measured using:

- Message read receipts (“blue ticks”) to assess content reach
- Task accomplishment rates, tracked using participants’ interactions (e.g., feedback, photo posting, voice messages) with tasks presented
- Interaction frequency, measured by the number of participant-generated responses or queries per module

Additionally, the moderator(s) will be responsible for documenting all engagement in an activity log during the intervention classes and also conduct separate inter-rater reliability checks on a portion of the engagement log. All interactions will be recorded by the moderator in an activity log. These metrics will serve as a proxy for determining participant’s access to and engagement with digital content (56) and will inform the conclusions of the qualitative feedback on the level of WhatsApp engagement in a nutrition intervention program.

##### 2.4.2.2 Knowledge retention

Tests using quizzes, polls, Google forms, and self-confidence assessment questions will be used to evaluate the knowledge retention rate of the participants. The tests will focus on the content of the class modules. A Google form knowledge retention assessment questionnaire will be administered, and participants’ audio and video feedback on the intervention modules will be used to assess how the participants have integrated the knowledge gained into their daily activities. This will reflect the effectiveness and efficiency of the intervention program regarding knowledge retention and behavioral change of the participants (57). The integration of these approaches will evaluate the impact on cognitive retention and the real application of knowledge acquired.

##### 2.4.2.3 Intervention acceptability

**Figure 4:**
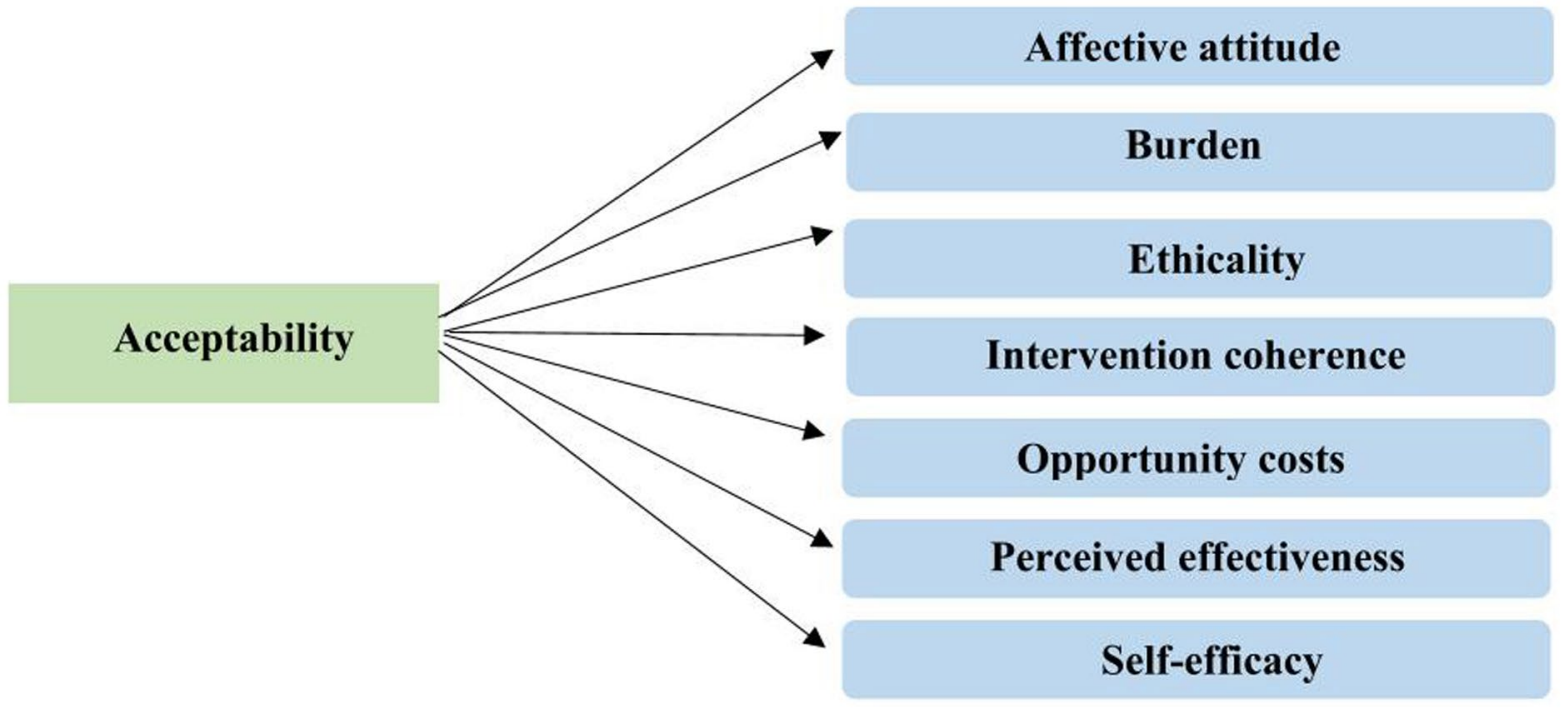
Theoretical framework of acceptability of health-related interventions.

The intervention acceptability metric based on the Theoretical Framework of Acceptability (TFA) developed by Sekhon et al. (58) as shown in Figure 4, will be used to determine the acceptability of the nutrition intervention. A questionnaire that captures the seven domains (affective attitude, burden, ethicality, intervention coherence, opportunity costs, perceived effectiveness, and self-efficacy) of acceptability regarding health-related intervention adapted from (59) will be used. The definition of the domains is adapted from Chen et al. (60) is shown in Table 3. Participants’ feedback on the questionnaire on the acceptability of the intervention will be used to determine the acceptability rate of the intervention.

**Table 3:** Definitions of theoretical framework of acceptability domains.

Domains	Definitions
Affective attitude	Participants feelings about the intervention
Burden	The perceived amount of effort that is required to participate in the intervention
Ethicality	The level at which the intervention fits into the value system of the participants
Intervention coherence	Participants’ comprehension level of the intervention
Opportunity costs	The level of compromises to participate in the intervention
Perceived effectiveness	The efficacy of the intervention
Self-efficacy	Perceived participants’ behavioral capacity

Source: Adapted from (60).

### 2.5 Data analysis

Descriptive statistics such as frequency and percentages will be used for the socio-demographic characteristics (such as age, gender, student status, and household size) and other outcome of measures (such as knowledge retention, intervention acceptability, and nutrition-related outcomes) that are categorical variables, while mean and standard deviation will be used for the continuous variables. To assess the possibility of bias resulting from missing data, we will conduct sensitivity analyses by carrying out complete case analyses. As opined by Braun and Clarke, this will ensure the robustness and credibility of our result (61). Mixed model analyses will be used to assess the intervention’s impact on skin carotenoid levels, anthropometrics, and diet diversity score (62, 63). The mixed model analysis was chosen because it accounts for repeated measures and can effectively address issues related to missing data. In these analyses, the intervention arms will be treated as the fixed effect so that the measure’s outcome change between the school gardening-only participants and school gardening and nutrition education participants can be assessed by the coefficients or estimated using the least square means. Time and intervention arm will be included as both main effects and interaction effects. The random effect will account for variability variations between groups that might affect the response. We will control for participant’s age, gender, household size, youth status (out-of-school vs. in-school status), community, and state, by including them as covariates in the adjusted models of the mixed model analysis. Mann–Whitney U Test will be used to compare the acceptability of the nutrition intervention between the groups. The analysis will be performed using IBM SPSS (version 23), and the statistical significance will be set at *p*-value <0.05.

As described by Braun and Clarke (61), the participants’ audio (sessions responses) and video recordings (tasks) from WhatsApp engagement will be analyzed using thematic analysis. We will use inductive coding to deduce the theme directly from the data. The convergent mixed method will be used to integrate the qualitative analysis with the quantitative results. This ensures a proper understanding of how participants experienced and engaged with the intervention.

## 3 Discussion

The study aims to improve the dietary habits of youths through a combined approach of school-based gardening of IVFs and digital delivery of nutrition education through WhatsApp. The study will fill the knowledge gap related to agricultural practices and nutritional knowledge of the youths, a population widely considered to influence future agriculture practices and food choices (64, 65). Furthermore, the study will provide relevant insights into designing and implementing innovative multi-level approaches that can improve dietary choices and enhance the health status of young people in the target region. Additionally, the study will serve as an evidence-based reference on the effectiveness of the strategies through the feasibility assessment of the approaches that would help in scaling sustainable nutrition programs and curbing malnutrition, thereby increasing the region’s health, social well-being, and economic progress.

The study will reveal how the combination of school-based gardening programs and mobile health technologies can impact the youths’ agricultural involvement and food habits. In addition, school garden programs have been regarded as one of the significant nutrition education tools as they connect experimental learning to food production and consumption (19, 66, 67). Likewise, this will involve growing IVFs through climate-smart agriculture practices to enhance knowledge of food habits and environmental management.

The integration of nutrition education through WhatsApp will bridge the gap between nutrition educators and the youths in low-income countries such as Nigeria; this approach is scalable for the promotion of food habits as there is rapid growth in internet access and mobile phone ownership among the youth (68–70). Furthermore, the emphasis on IVFs will aid in achieving the goal of food sovereignty and sustainability (13, 14, 71).

The study would be interpreted within the context of cultural, environmental, and technological concept of southwest Nigeria, where the intervention tailored to align with the local agricultural practices, dietary habits, and mobile technology usage.

### 3.1 Barriers, constraints, and mitigation strategies

To ensure the sustainability of the intervention beyond the study period, it is crucial to secure ongoing commitment from staff and students. In addition to funding, school gardening relies on sustained participation, as interest may fade over time. To address this, we will engage agricultural teachers at the selected schools and key community leaders during the intervention to maintain the program’s momentum. Furthermore, the *Roots and Greens* curriculum will be made publicly available after the study to support the long-term use of the gardening program. The researchers acknowledge potential biases associated with a non-randomized study design, such as selection bias and reduced internal validity. However, this design was chosen to take advantage of participants already being organized into natural groups based on their access to mobile phones, which will help minimize logistical challenges. Moreover, non-randomized designs offer external validity which often closely reflect real-life settings, enabling researchers to study how interventions operate in typical community or school environments. Furthermore, we acknowledge that the purposive sampling techniques may limit generalization, however we took steps to reduce bias and promote representativeness in the sample by stratifying recruitment to ensure diversity among youth status (in-school and out-of-school), geography (urban and rural), and state representation in Southwest Nigeria. Moreover, we intentionally oversampled certain groups (e.g., out-of-school youth) to ensure sufficient coverage of otherwise hard-to-reach sub-populations. These strategies enhance the practical generalizability of the findings to similar real-world environments (72).

While the study involves self-reported outcomes and other measures, enumerators will be trained before the data collection period to uphold participant confidentiality to encourage honest responses and reduce response bias. To further minimize response bias in self-reported data, confidentiality assurances will be emphasized during the consent process. Moreover, the proposed analysis, mixed method, will control confounding factors to minimize the differences in participants’ characteristics and account for variability in the data. Additionally, participant dropout is anticipated; however, oversampling will address attrition, and the mixed model analysis will address missing data. The context-specific approach serves to enhance the relevance of an intervention and its applicability in a given study setting the generalizability may be limited for those regions with totally different infrastructures or sociocultural contexts. Nevertheless, the knowledge acquired provides a valuable learning base for designing comparable interventions in other contexts, still through appropriate contextual adaptations. The study is designed to assess the immediate impact of the intervention on nutritional knowledge, dietary habits, and participation in agricultural activities. The longer-term effects of the intervention, particularly about sustainable nutritional changes and health indicators, will be better understood through future longitudinal or follow-up studies. Future studies should put the study findings into perspective and further clarify the effectiveness of the interventions over time.

### 3.2 Implications for practice involvement, policy development, and future research directions

The demonstrated effectiveness of the study could serve as a model for integrating agriculture and nutrition education into school curricula to enhance agricultural practices and food habits. Additionally, this model holds promise for adaptation in non-school settings to reach out-of-school youths through community-based programs, vocational training centers, or digital platforms, thereby extending its impact to a broader population of young people. This has the potential to significantly impact how schools in low-income countries approach agricultural and nutrition education. The success of this model can have far-reaching implications for agricultural and nutrition education policies in Nigeria and other low-income countries, ultimately contributing to a reduction in food insecurity. In addition, the delivery of nutrition education through digital platforms will widen the coverage of the target audience by including parents and community members, reducing the impacts of ignorance and cultural apathy on the consumption of IVFs.

Further research could determine the intervention’s acceptability, scalability, and adaptation across populations and regions. A randomized longitudinal study can be carried out to determine the long-term impacts of this intervention on agricultural practices, dietary behaviors, and nutritional status. Furthermore, studies involving other digital platforms other than WhatsApp can be carried out to establish the sustainability of mobile health nutrition education intervention.

## Data Availability

The original contributions presented in the study are included in the article, further inquiries can be directed to the corresponding author.

## References

[1] Ettinger AK Risser L Rahman S Rigas D Abromitis R Stokes LR . Defining and measuring child and youth thriving: a scoping review. Pediatrics. (2022) 150:6902. doi: 10.1542/peds.2022-056902, PMID: 36239092

[2] Ministry of Youth and Sports Development N. (2019). Second National Youth Policy Document of the Federal Republic of Nigeria 2009. Available online at: https://prb.org/wp-content/uploads/2019/06/Nigeria_2009_National_Youth_Policy.pdf (Accessed April 4, 2025).

[3] Akanni Akinyemi. (2023). The conversation. Nigeria’s growing population can be an advantage, with better data and a policy focus on young people. Available online at: https://theconversation.com/nigerias-growing-population-can-be-an-advantage-with-better-data-and-a-policy-focus-on-young-people-209530 (Accessed November 8, 2024).

[4] Olatona FA Ogide PI Abikoye ET Ilesanmi OT Nnoaham KE. Dietary diversity and nutritional status of adolescents in Lagos, Nigeria. J Family Med Prim Care. (2023) 12:1547–54. doi: 10.4103/jfmpc.jfmpc_1783_22, PMID: 37767409 PMC10521850

[5] Adeomi AA Fatusi A Klipstein-Grobusch K. Food security, dietary diversity, dietary patterns and the double burden of malnutrition among school-aged children and adolescents in two Nigerian states. Nutrients. (2022) 14:789. doi: 10.3390/nu14040789, PMID: 35215439 PMC8875779

[6] Abubakar HA Shahril MR Mat S. Nutritional status and dietary intake among Nigerian adolescent: a systematic review. BMC Public Health. (2024) 24:1–13. doi: 10.1186/s12889-024-19219-w 38956547 PMC11221175

[7] Ogbuabor DC. Magnitude and determinants of food insecurity among pregnant women in Nigeria: evidence from a Nationwide cross-sectional survey. Int J Med Health Dev. (2024) 29:227–37. doi: 10.4103/ijmh.ijmh_13_24

[8] Obayelu O. Food insecurity in urban slums: evidence from Ibadan Metropolis, Southwest Nigeria. Journal for the advancement of. Dev Econ. (2018) 7:1–17. doi: 10.32873/unl.dc.jade7.1.2

[9] Orjiakor EC Adediran A Ugwu JO Nwachukwu W. Household living conditions and food insecurity in Nigeria: a longitudinal study during COVID-19 pandemic. BMJ Open. (2023) 13:e066810. doi: 10.1136/bmjopen-2022-066810, PMID: 36604138 PMC9826925

[10] Otekunrin OA Otekunrin OA. Exploring dietary diversity, nutritional status of adolescents among farm households in Nigeria: do higher commercialization levels translate to better nutrition? Nutr Food Sci. (2023) 53:500–20. doi: 10.1108/NFS-03-2022-0104

[11] Salam RA Hooda M Das JK Arshad A Lassi ZS Middleton P . Interventions to improve adolescent nutrition: a systematic review and Meta-analysis. J Adolesc Health. (2016) 59:S29–39. doi: 10.1016/j.jadohealth.2016.06.022, PMID: 27664593 PMC5026685

[12] Shapu RC Ismail S Lim PY Ahmad N Garba H Njodi IA. Effectiveness of triple benefit health education intervention on knowledge, attitude and food security towards malnutrition among adolescent girls in Borno state, Nigeria. Food Secur. (2022) 11:130. doi: 10.3390/foods11010130, PMID: 35010256 PMC8750727

[13] Kennedy G Kanter R Chotiboriboon S Covic N Delormier T Longvah T . Traditional and indigenous fruits and vegetables for food system transformation. Curr Dev Nutr. (2021) 5:nzab092. doi: 10.1093/cdn/nzab092, PMID: 34423230 PMC8373596

[14] Cogill B. Contributions of indigenous vegetables and fruits to dietary diversity and quality. Acta Hortic. (2015) 1102:213–28. doi: 10.17660/ActaHortic.2015.1102.27, PMID: 37111230

[15] Kiyimba T Yiga P Bamuwamye M Vandamme E Ogwok P Van Der Schueren B . (2023). Exploring indigenous fruits and vegetables with potential cardiometabolic health benefits: understanding barriers and facilitators to consumption. doi: 10.21203/rs.3.rs-3107648/v1 PMC12706100 41409765

[16] Tanimonure VA Naziri D Codjoe SNA Ayanwale AB. Underutilised indigenous vegetables for household dietary diversity in Southwest Nigeria. Agriculture (Switzerland). (2021) 11:1064. doi: 10.3390/agriculture11111064, PMID: 40278680

[17] Corrochano D Ferrari E López-Luengo MA Ortega-Quevedo V. Educational gardens and climate change education: an analysis of Spanish preservice teachers’ perceptions. Educ Sci (Basel). (2022) 12:275. doi: 10.3390/educsci12040275, PMID: 40278680

[18] Duncan DW Collins A Fuhrman NE Knauft DA Berle DC. The impacts of a school garden program on urban middle school youth. J Agric Educ. (2016) 57:174–85. doi: 10.5032/jae.2016.04174

[19] Holloway TP Dalton L Hughes R Jayasinghe S Patterson KAE Murray S . School gardening and health and well-being of school-aged children: a realist synthesis. Nutrients. (2023) 15:1190. doi: 10.3390/nu15051190, PMID: 36904189 PMC10005652

[20] International Union of Nutritional Sciences (2024). Traditional and indigenous food systems and nutrition. Available online at: https://iuns.org/taskforces/traditional-and-indigenous-food-systems-and-nutrition (Accessed December 10, 2024).

[21] Adewoyin A Torimiro A Oyedele A. Assessment of perceived knowledge and consumption frequency of underutilized indigenous vegetables (UIVs) among the rural youth in Osun state, Nigeria. Acta Hortic. (2019) 1238:177–84. doi: 10.17660/ActaHortic.2019.1238.18

[22] Asekenye C Alele PE Ogwang PE Olet EA. Frequency of consumption of green leafy vegetables and prevalence of Hyperglycaemia in Ankole and Teso sub-regions of Uganda. J Clin Transl Res. (2023) 9:398–413. doi: 10.18053/jctres.09.202306.23-00096

[23] Awuni V Adjei Yeboah G Mohammed Misbah J Zakaria A Amagloh FK. Nutrition knowledge, cooking practices, and consumption of indigenous leafy vegetables among households in Sagnarigu municipality, Ghana. Ghana J Sci. (2022) 8:24–32. Available online at: https://www.ajol.info/index.php/gjstd/article/view/239582

[24] Emmanuel R Read UM Grande AJ Harding S. Acceptability and feasibility of community gardening interventions for the prevention of non-communicable diseases among indigenous populations: a scoping review. Nutrients. (2023) 15:791. doi: 10.3390/nu15030791, PMID: 36771495 PMC9921708

[25] Kodzwa M Masvaya N. Optimization of African indigenous vegetables production in sub Saharan Africa: a review. CABI Agric Biosci. (2023) 4:1–10. doi: 10.1186/s43170-023-00184-0, PMID: 40306641

[26] Ajani OA Khoalenyane NB. Using WhatsApp as a tool of learning: a systemic literature review of prospects and challenges. Int J Innov Technol Soc Sci. (2023) 3:8025. doi: 10.31435/rsglobal_ijitss/30092023/8025

[27] Usman YD (2022). BA Students’ usage of WhatsApp instant messenger as a supporting tool for learning in Kaduna state, Nigeria Yunusa Dangara Usman Federal Road Safety Corps, Kaduna, Nigeria Aishatu Bukar local education authority, Kwali Area Council Abuja, Nigeria. Available online at: https://eric.ed.gov/?id=EJ1378017 (Accessed December 10, 2024).

[28] Ochieng BM Smith L Orton B Hayter M Kaseje M Wafula CO . Perspectives of adolescents, parents, service providers, and teachers on Mobile phone use for sexual reproductive health education. Sociol Sci. (2022) 11:196. doi: 10.3390/socsci11050196

[29] Sahu M Ashoo G Joshi A. Role of Mobile phone Technology in Health Education in Asian and African countries: a systematic review. Int J Electron Healthc. (2014) 7:269–86. doi: 10.1504/IJEH.2014.064327, PMID: 25161104

[30] Amosu AM Kukoyi OB. Knowledge and attitude of Nigerian undergraduates on healthy diet: outcome of a nutrition education and Mobile phone communication intervention. Texila. Int J Public Health. (2020) 8:1–9. doi: 10.21522/TIJPH.2013.08.02.Art001

[31] Sohail D. Using the WhatsApp social media application for active learning. J Educ Technol Syst. (2020) 49:239–49. doi: 10.1177/0047239520928307, PMID: 40297624

[32] Suárez-Lantarón B Deocano-Ruíz Y García-Perales N Castillo-Reche IS. The educational use of WhatsApp. Sustainability. (2022) 14:510. doi: 10.3390/su141710510

[33] Trude ACB Martins RC Martins-Silva T Blumenberg C Carpena MX Del-Ponte B . A WhatsApp-based intervention to improve maternal social support and maternal–child health in southern Brazil: the text-message intervention to enhance social support (TIES) feasibility study. Inquiry (United States). (2021) 58:510. doi: 10.1177/00469580211048701 PMC8504647 34619999

[34] Mancone S Corrado S Tosti B Spica G Diotaiuti P. Integrating digital and interactive approaches in adolescent health literacy: a comprehensive review. Front Public Health. (2024) 12:1387874. doi: 10.3389/fpubh.2024.1387874, PMID: 39444982 PMC11496154

[35] Onyekwere A. Effects of youth unemployment on the Nigerian society: the need for resourceful intervention. Int J Soc Sci Manag Res. (2021) 7:24–46. Available online at: https://www.iiardjournals.org/get/IJSSMR/VOL.%207%20NO.%201%202021/EFFECTS%20OF%20YOUTH%20UNEMPLOYMENT.pdf

[36] National Bureau of Statistics. (2023). Nigeria labour force survey Q2 2023. Nigeria. Available online at: https://www.nigerianstat.gov.ng/elibrary/read/1241429 (Accessed April 8, 2025).

[37] World Bank Group Data. (2025). International Labour Organization. “ILO modelled estimates and projections database (ILOEST)” ILOSTAT. Unemployment, youth Total (% of Total labor force ages 15–24) (modeled ILO estimate) - Nigeria. Available online at: https://data.worldbank.org/indicator/SL.UEM.1524.ZS?locations=NG&utm_source (Accessed April 9, 2025).

[38] Federal Ministry of Youth and Sports Development. (2021). Nigerian youth employment action plan (NIYEAP) 2021–2024. Available online at: https://faolex.fao.org/docs/pdf/nig229329.pdf (Accessed April 9, 2025).

[39] Weigand H Kish L. Survey sampling. John Wiley & Sons, Inc., New York, London 1965, IX + 643 S., 31 Abb., 56 tab., Preis 83 s. Biom J. (1968) 10:88–9. doi: 10.1002/bimj.19680100122

[40] Calculator.net. (2025). Available online at: https://www.calculator.net/sample-size-calculator.html (Accessed March 30, 2025).

[41] de Andrade Hovadick AC Cardoso MA. Family-based WhatsApp intervention to promote healthy eating behaviors among Amazonian school children: protocol for a randomized controlled trial. JMIR Res Protoc. (2024) 13:e54446. doi: 10.2196/54446, PMID: 38373039 PMC10912988

[42] Adisa Ojerinde Famakinwa. Factors influencing youths’ utilization of underutilized indigenous vegetable innovations as a livelihood strategy in southwestern Nigeria. Trop Agric Res Ext. (2017) 20:105–14. doi: 10.4038/tare.v20i3-4.5396

[43] Stadlmayr B Trübswasser U Mcmullin S Karanja A Wurzinger M Hundscheid L . Factors affecting fruit and vegetable consumption and purchase behavior of adults in sub-Saharan Africa: a rapid review. Front Nutr. (2023) 10:1113013. doi: 10.3389/fnut.2023.1113013, PMID: 37113298 PMC10126510

[44] Hochberg K Becker S Louis M Klein P Kuhn J. Using smartphones as experimental tools—a follow-up: cognitive effects by video analysis and reduction of cognitive load by multiple representations. J Sci Educ Technol. (2020) 29:303–17. doi: 10.1007/s10956-020-09816-w

[45] Kennedy JL Christensen CG Maxon TS Gerard SN Garcia EB Kook JF . The efficacy of digital media resources in improving Children’s ability to use informational text: an evaluation of Molly of Denali from PBS KIDS. Am Educ Res J. (2022) 59:1194–228. doi: 10.3102/00028312221113326

[46] Adekoya AE Adenikinju AF Olubusoye OE Oyeranti OA Otekunrin OA Ogunbayo IE . Household food insecurity and cooking energy access in Nigeria: a panel data approach. Energy Nexus. (2023) 12:100242. doi: 10.1016/j.nexus.2023.100242, PMID: 40309691

[47] Suzanne PMPR Lucia LKPR Marilyn STPR Lindsay HAPR. Evaluation of validity of items for a food behavior checklist. J Am Diet Assoc. (2001) 101:751–61. doi: 10.1016/S0002-8223(01)00189-4, PMID: 11478471

[48] Mainvil LA Lawson RP Horwath CC Mckenzie JE Reeder AI. Validated scales to assess adult self-efficacy to eat fruits and vegetables. Am J Health Promot. (2009) 23:210–7. doi: 10.4278/ajhp.061221154, PMID: 19149427

[49] Byrd-Bredbenner C Wheatley V Schaffner D Bruhn C Blalock L Maurer J. Development of food safety psychosocial questionnaires for young adults. J Food Sci Educ. (2007) 6:30–7. doi: 10.1111/j.1541-4329.2007.00021.x

[50] Swindale A Bilinsky P. (2006). Household dietary diversity score (HDDS) for measurement of household food access: Indicator guide (version 2). Available online at: https://www.fantaproject.org/sites/default/files/resources/HDDS_v2_Sep06_0.pdf (Accessed April 8, 2025).

[51] FAO (2011). Food and agriculture Organization of the United Nations. Guidelines for measuring household and individual dietary diversity. Italy. Available online at: https://openknowledge.fao.org/handle/20.500.14283/i1983e (Accessed November 8, 2024).

[52] WHO. (2024). Obesity and overweight. Available online at: https://www.who.int/news-room/fact-sheets/detail/obesity-and-overweight (Accessed October 8, 2024).

[53] Ermakov IV Whigham L Redelfs A Jahns LA Stookey J Berstein PS . (2016). “Skin carotenoids as biomarker for vegetable and fruit intake: validation of the reflection-spectroscopy based “veggie meter”. in *Federation of American Societies for Experimental Biology conference, 2016, San Diego, California*. 30:896.19.

[54] Sharma P Singh AK Leiva V Martin-Barreiro C Cabezas X. Modern multivariate statistical methods for evaluating the impact of WhatsApp on academic performance: methodology and case study in India. Appl Sci. (2022) 12:6141. doi: 10.3390/app12126141, PMID: 40278680

[55] Zulkanain NA Miskon S Abdullah NS. An adapted pedagogical framework in utilizing WhatsApp for learning purpose. Educ Inf Technol. (2020) 25:2811–22. doi: 10.1007/s10639-019-10096-0

[56] Singer B Walsh CM Gondwe L Reynolds K Lawrence E Kasiya A. WhatsApp as a medium to collect qualitative data among adolescents: lessons learned and considerations for future use. Gates Open Res. (2020) 4:130. doi: 10.12688/gatesopenres.13169.1

[57] Vander Wyst KB Vercelli ME O’Brien KO Cooper EM Pressman EK Whisner CM. A social media intervention to improve nutrition knowledge and behaviors of low income, pregnant adolescents and adult women. PLoS One. (2019) 14:e0223120. doi: 10.1371/journal.pone.0223120, PMID: 31647852 PMC6812786

[58] Sekhon M Cartwright M Francis JJ. Development of a theory-informed questionnaire to assess the acceptability of healthcare interventions. BMC Health Serv Res. (2022) 22:279. doi: 10.1186/s12913-022-07577-3, PMID: 35232455 PMC8887649

[59] Haydon HM Major T Kelly JT Catapan S De C Caffery LJ Smith AC . Development and validation of the digital health acceptability questionnaire. J Telemed Telecare. (2023) 29:8S–15S. doi: 10.1177/1357633X231202279, PMID: 38007698

[60] Chen E Moracco KE Kainz K Muessig KE Tate DF. Developing and validating a new scale to measure the acceptability of health apps among adolescents. Digit Health. (2022) 8:8. doi: 10.1177/20552076211067660, PMID: 35154802 PMC8832596

[61] Braun V Clarke V. Using thematic analysis in psychology. Qual Res Psychol. (2006) 3:77–101. doi: 10.1191/1478088706qp063oa

[62] West TB Welch KB Galecki AT. Linear mixed models: A practical guide using statistical software. 1st ed. New York: Chapman and Hall/CRC (2006). doi: 10.1201/9781003181064

[63] Yang RC. Towards understanding and use of mixed-model analysis of agricultural experiments. Can J Plant Sci. (2010) 90:605–27. doi: 10.4141/CJPS10049

[64] Dominic G James S. Youth and food systems transformation. Front Sustain Food Syst. (2020) 4:1–15. doi: 10.3389/fsufs.2020.00101, PMID: 40309010

[65] Mariaelena H Indika A Evan B Anna K Catherine M Paola T . Knowledge networks to support youth engagement in sustainable food systems. Front Sustain Food Syst. (2022) 6:1–14. doi: 10.3389/fsufs.2022.867344, PMID: 40309010

[66] Parmer SM Salisbury-Glennon J Shannon D Struempler B. School gardens: an experiential learning approach for a nutrition education program to increase fruit and vegetable knowledge, preference, and consumption among second-grade students. J Nutr Educ Behav. (2009) 41:212–7. doi: 10.1016/j.jneb.2008.06.002, PMID: 19411056

[67] Chan CL Tan PY Gong YY. Evaluating the impacts of school garden-based Programmes on diet and nutrition-related knowledge, attitudes and practices among the school children: a systematic review. BMC Public Health. (2022) 22:1251. doi: 10.1186/s12889-022-13587-x, PMID: 35751069 PMC9233338

[68] French ML Christensen JT Estabrooks PA Hernandez AM Metos JM Marcus RL . Evaluation of the effectiveness of a bilingual nutrition education program in partnership with a mobile health unit. Nutrients. (2024) 16:618. doi: 10.3390/nu16050618, PMID: 38474746 PMC10934044

[69] Yalinie K Jill H Ananya B Anatoliy G Barkha P Jennifer S. Social media interventions for nutrition education among adolescents: scoping review. JMIR Pediatr Parent. (2023) 6:e36132. doi: 10.2196/36132, PMID: 37471119 PMC10401194

[70] Sindi A Stanfield J Sheikh A. Technology in education: attitudes towards using technology in nutrition education. Int J Adv Comput Sci Appl. (2021) 12:59–71. doi: 10.14569/IJACSA.2021.0120208

[71] Wolfgang B Susanne H-K Zoltan F Silke S. The role of indigenous vegetables to improve food and nutrition security: experiences from the project HORTINLEA in Kenya (2014–2018). Front Sustain Food Syst. (2022) 6:806420. doi: 10.3389/fsufs.2022.806420

[72] Yang C Fridgeirsson EA Kors JA Reps JM Rijnbeek PR. Impact of random oversampling and random Undersampling on the performance of prediction models developed using observational health data. J Big Data. (2024) 11:7. doi: 10.1186/s40537-023-00857-7

